# Reduced anoctamin 7 (ANO7) expression is a strong and independent predictor of poor prognosis in prostate cancer

**DOI:** 10.20892/j.issn.2095-3941.2019.0324

**Published:** 2021-02-15

**Authors:** Andreas Marx, Lena Koopmann, Doris Höflmayer, Franziska Büscheck, Claudia Hube-Magg, Stefan Steurer, Till Eichenauer, Till S. Clauditz, Waldemar Wilczak, Ronald Simon, Guido Sauter, Jakob R. Izbicki, Hartwig Huland, Hans Heinzer, Markus Graefen, Alexander Haese, Thorsten Schlomm, Christian Bernreuther, Patrick Lebok, Sarah Bonk

**Affiliations:** 1 Institute of Pathology, Klinikum Fürth, Fürth 90766, Germany; 2 Institute of Pathology, University Medical Center Hamburg-Eppendorf, Hamburg 20246, Germany; 3 Department of Urology, University Medical Center Hamburg-Eppendorf, Hamburg 20246, Germany; 4 General, Visceral and Thoracic Surgery Department and Clinic, University Medical Center Hamburg-Eppendorf, Hamburg 20246, Germany; 5 Martini-Clinic, Prostate Cancer Center, University Medical Center Hamburg-Eppendorf, Hamburg 20246, Germany; 6 Department of Urology, Charité – Universitätsmedizin Berlin, Berlin 10117, Germany

**Keywords:** ANO7, immunohistochemistry, prognosis, prostate cancer

## Abstract

**Objective:**

Anoctamin 7 (ANO7) is a calcium^2+^-dependent chloride ion channel protein. Its expression is restricted to prostate epithelial cells. The exact function is unknown. This study aimed to analyze ANO7 expression and its clinical significance in prostate cancer (PCa).

**Methods:**

ANO7 expression was assessed by immunohistochemistry in 17,747 clinical PCa specimens.

**Results:**

ANO7 was strongly expressed in normal prostate glandular cells but often less abundant in cancer cells. ANO7 staining was interpretable in 13,594 cancer tissues and considered strong in 34.4%, moderate in 48.7%, weak in 9.3%, and negative in 7.6%. Reduced staining was tightly linked to adverse tumor features [high classical and quantitative Gleason grade, lymph node metastasis, advanced tumor stage, high Ki67 labeling index, positive surgical margin, and early biochemical recurrence (*P* < 0.0001 each)]. The univariate Cox hazard ratio for prostate-specific antigen (PSA) recurrence after prostatectomy in patients with negative *vs.* strong ANO7 expression was 2.98 (95% confidence interval 2.61–3.38). The prognostic impact was independent of established pre- or postoperatively available parameters (*P* < 0.0001). Analysis of annotated molecular data showed that low ANO7 expression was linked to *TMPRSS2:ER*G fusions (*P* < 0.0001), elevated androgen receptor expression (*P* < 0.0001), as well as presence of 9 of 11 chromosomal deletions (*P* < 0.05 each). A particularly strong association of low ANO7 expression with phosphatase and tensin homolog (PTEN) deletion may indicate a functional relationship with the PTEN/AKT pathway.

**Conclusions:**

These data identify reduced ANO7 protein expression as a strong and independent predictor of poor prognosis in PCa. ANO7 measurement, either alone or in combination, might provide clinically useful prognostic information in PCa.

## Introduction

With an estimated 1.3 million new cases worldwide in 2018, prostate cancer (PCa) is the most common cancer in men. Despite a rather indolent clinical course of most PCa, this disease still represents the third most common cause of cancer-related death^1^. Treatment options vary from radical surgery to active surveillance and are chosen based on the perceived cancer aggressiveness. Gleason grade and tumor extent on biopsies, preoperative prostate-specific antigen (PSA), and clinical stage are currently the established pretreatment parameters. Although these data are statistically powerful, they do not always allow optimal treatment decisions in retrospect. Thus it is hoped that new molecular biomarkers will enable a precise prediction of PCa aggressiveness.

Anoctamins are a family of at least 10 calcium^2+^-dependent chloride ion channels with physiological roles in epithelial and other cell types^2^. Anoctamin 7 (ANO7) is of particular interest for PCa biology. It was originally termed NGEP (new gene expressed in prostate), as its expression is virtually restricted to prostate epithelial cells^3^. Early immunohistochemical studies revealed that ANO7 is a transmembrane cell junction protein strongly expressed at the apical pole and on lateral surfaces of the epithelial cells of prostate glands^4^, but its exact function is still unknown. ANO7 resides at 2q37, a genomic region that has been associated with PCa risk before^5^. Immunohistochemical analyses on 120–160 samples of normal and cancerous prostate tissues found that ANO7 expression is retained during PCa development but decreases with progression to high Gleason grade^6,7^, suggesting a role of ANO7 loss for PCa biology. In conflict with these data, a recent analysis of ANO7 messenger RNA (mRNA) expression from 289 PCa patients included in The Cancer Genome Atlas project^8^ suggested that increased ANO7 expression might indicate poor patient outcome^9^.

These contradictory findings prompted us to evaluate the potential prognostic impact of ANO7 expression in PCa by using our tissue microarray (TMA) comprising tumor samples from more than 17,000 individual patients.

## Materials and methods

### Patients

The 17,747 patients had prostatectomy between 1992 and 2015 (Department of Urology and the Martini Clinic at the University Medical Center Hamburg-Eppendorf). PSA recurrence was defined as the time point when postoperative PSA was at least 0.2 ng/mL and increasing at subsequent measurements. **Supplementary Table S1** summarizes the patient characteristics on the TMA. Quantitative Gleason grading supplemented classical Gleason categories^10^. The TMA was produced with a single 0.6-mm core taken from a tumor-containing tissue block from each patient^11^. The attached molecular database included data on Ki67 labeling index (Ki67LI)^12^, erythroblast transformation-specific-related gene (ERG) protein expression, and ERG rearrangement analysis by fluorescence *in situ* hybridization (FISH)^13,14^, as well as deletion status of 3p13 [forkhead box protein F1 (FOXP1)]^15^, 5q21 [chromodomain-helicase-DNA-binding protein 1 (CHD1)]^16^, 6q15 [mitogen-activated protein kinase kinase kinase 7 (MAP3K7)]^17^, 8p21^18^, 10q23 [phosphatase and tensin homolog (PTEN)]^19^, 12p13^20^, 12q24^21^, 13q14^22^, 16q24^23^, 17p13^24^, and 18q21^25^. Archived diagnostic leftover tissues were used in accordance with the local laws (HmbKHG, §12,1) and approved by the local ethics committee (Ethics commission Hamburg, WF-049/09). All work has been carried out in compliance with the Helsinki Declaration.

## Immunohistochemistry

Freshly cut TMA sections were stained on one day and in one experiment. Slides were deparaffinized and exposed to heat-induced antigen retrieval for 5 min at 121 °C in pH 7.8 Tris-EDTA buffer. Primary antibody specific for ANO7 (rabbit polyclonal antibody, Sigma-Aldrich, St. Louis, MO, USA, HPA035730; dilution 1:900) was applied at 37 °C for 60 min. Bound antibody was then visualized using the EnVision Kit (Dako, Glostrup, Denmark) according to the manufacturer’s directions. All tissues were analyzed by one trained pathologist. Anti-ANO7 showed cytoplasmic staining and the staining intensity (0, 1+, 2+, 3+) as well as the percentage of stained cells were recorded for each tissue spot. A final score was built from these two parameters as follows: lack of any staining (intensity 0) was considered “negative”, 1+ staining in ≤ 70% of tumor cells or 2+ staining in ≤ 30% of tumor cells was considered “weak”, 1+ staining in > 70% of tumor cells or 2+ staining in > 30% but ≤ 70% of tumor cells or 3+ staining in ≤ 30% of tumor cells was considered “moderate”, and 2+ staining in > 70% of tumor cells or 3+ staining in > 30% of tumor cells was considered “strong”.

### Statistics

Contingency tables and χ² tests were used to examine associations between molecular and histopathological tumor parameters. Kaplan–Meier survival curves were calculated, and the log-rank test was applied to detect differences between groups. Cox proportional hazards regression analysis was performed to test for independence between pathological, molecular, and clinical variables. Calculations were done with JMP 12 (SAS, Cary, NC, USA). All conducted analyses are referred to the overall *P* values.

## Results

A total of 67.6% of 17,747 tumor samples were interpretable in the TMA analysis. Reasons for non-informative cases (*n* = 4,153; 32.4%) included lack of tissue samples or absence of unequivocal cancer tissue in the TMA spot.

### ANO7 expression in normal and cancerous prostate tissues

**Figure 1:**
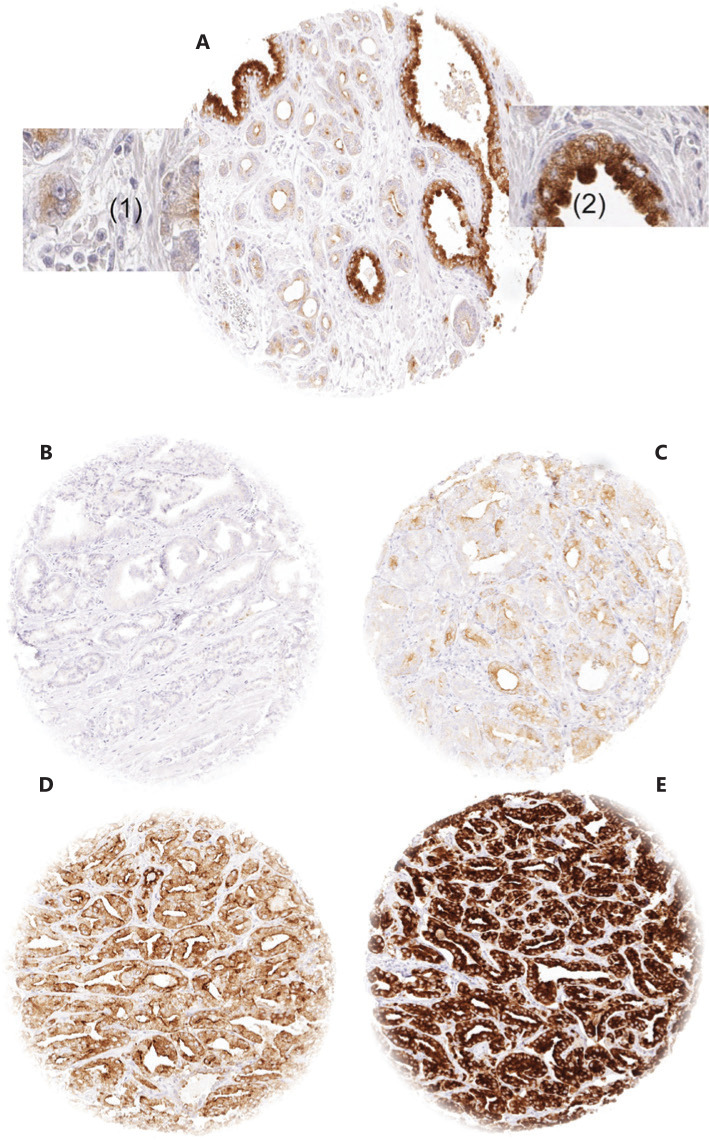
Examples of anoctamin 7 (ANO7) staining: (A) ANO7 staining of cancerous (1) and normal prostate gland (2) in a single tissue microarray (TMA) spot. (B–E) cancer spots with (B) lack of staining, (C) weak staining, (D) moderate staining, and (E) strong staining. Spot size 0.6 mm at 100× and 400× of origin.

**Figure 2:**
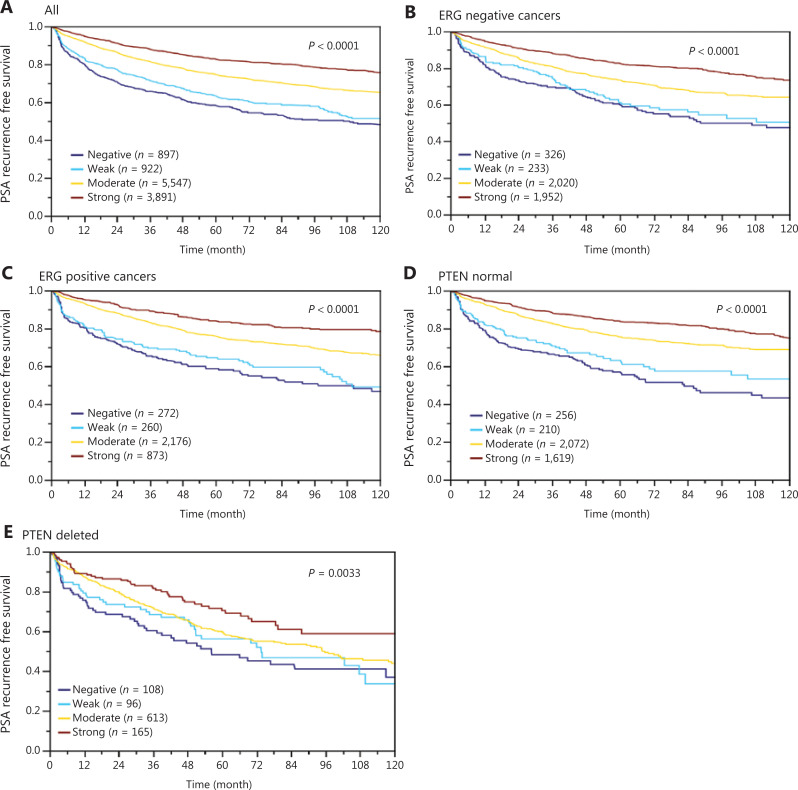
Association between anoctamin 7 (ANO7) expression and biochemical recurrence in (A) all cancers, (B) the erythroblast transformation-specific-related gene (ERG)-negative subset, (C) the ERG-positive subset, (D) phosphatase and tensin homolog (*PTEN*) normal cancers, and (E) *PTEN*-deleted cancers.

In normal prostate glands, luminal and basal cells stained moderately to strongly positive for ANO7. In PCa, cytoplasmic staining was considered strong in 34.4%, moderate in 48.7%, weak in 9.3%, and negative in 7.6% of 13,594 interpretable tumors. Representative images of ANO7 staining are given in **Figure 1**. Lost or reduced ANO7 staining showed significant associations with adverse tumor features. Low ANO7 expression was linked to advanced tumor stage (*P* < 0.0001), high classical and quantitative Gleason grade (*P* < 0.0001 each), presence of lymph node metastasis (*P* < 0.0001), high preoperative PSA level (*P* < 0.0001), and positive surgical margin (*P* < 0.0001; **Table 1**). Low ANO7 staining was also strongly linked to early biochemical recurrence (*P* < 0.0001, **Figure 2A**).

**Table 1:** ANO7 expression and prostate cancer phenotype

Parameter	*n*	Fraction of ANO7 expression (%)				*P*
		Negative	Weak	Moderate	Strong	
All cancers	13,594	7.6	9.3	48.7	34.4	
Tumor stage						< 0.0001
pT2	8889	5.5	7.0	47.4	40.2	
pT3a	2984	9.8	11.0	52.1	27.1	
pT3b-pT4	1668	14.5	18.5	49.6	17.3	
Gleason grade						< 0.0001
≤ 3+3	2730	4.9	5.6	46.2	43.3	
3+4	7372	5.3	7.7	49.8	37.3	
3+4 Tert.5	614	7.5	12.2	47.4	32.9	
4+3	1292	14.5	12.8	49.6	23.1	
4+3 Tert.5	898	13.0	18.3	50.8	17.9	
≥ 4+4	591	24.0	18.8	44.8	12.4	
Quantitative Gleason grade						
≤ 3+3	2730	4.9	5.6	46.2	43.3	< 0.0001
3+4 ≤ 5%	1927	3.9	5.3	46.4	44.4	
3+4 (6%–10%)	1845	4.3	6.0	50.4	39.3	
3+4 (11%–20%)	1614	4.8	7.7	51.4	36.1	
3+4 (21%–30%)	813	6.8	11.6	51.4	30.3	
3+4 (31%–49%)	663	8.9	8.9	53.4	28.8	
3+4 Tert.5	614	7.5	12.2	47.4	32.9	
4+3 (50%–60%)	556	9.9	10.4	50.0	29.7	
4+3 Tert.5	898	13.0	18.3	50.8	17.9	
4+3 ≥ 61%	559	17.4	12.9	50.8	19.0	
≥ 4+4	511	23.3	17.0	46.8	12.9	
Lymph node metastasis						< 0.0001
N0	8150	7.9	9.7	48.9	33.4	
N+	864	17.4	19.9	45.7	17.0	
Preoperative PSA level (ng/mL)						< 0.0001
< 4	1657	8.8	8.6	49.3	33.3	
4–10	8140	6.3	8.3	48.2	37.2	
10–20	2817	8.9	10.7	49.7	30.6	
> 20	898	12.6	15.0	48.6	23.8	
Surgical margin						< 0.0001
Negative	10,922	6.9	8.5	48.5	36.0	
Positive	2624	10.1	12.5	49.4	28.0	

ANO7, anoctamin 7; PSA, prostate-specific antigen.

### ANO7, TMPRSS2:ERG fusion status, and androgen receptor expression

**Figure 3:**
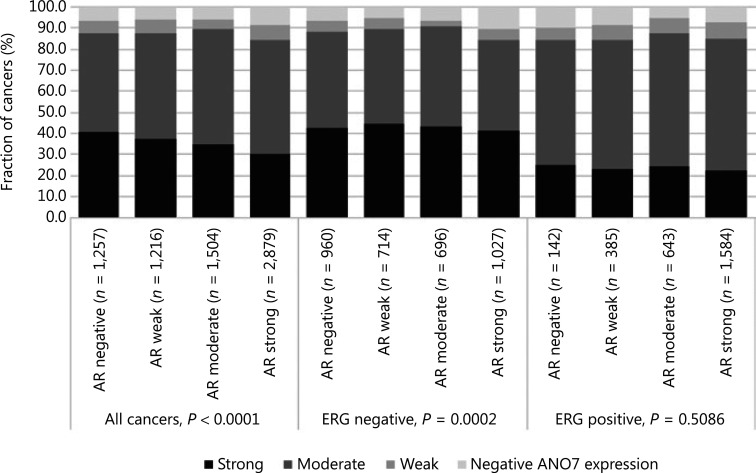
Anoctamin 7 (ANO7) staining and androgen receptor (AR) expression in all cancers, the erythroblast transformation-specific-related gene (ERG)-negative, and ERG-positive subsets.

Data from 5656 tumors with *ERG* FISH and immunohistochemistry (IHC) were available and showed concordant results in 95.5% of cancers. Strong ANO7 staining was less prevalent in cancers harboring *TMPRSS2:ERG* rearrangements (24%) and ERG expression (25%) than in cancer lacking *ERG* fusions (43%) or ERG expression (45%, *P* < 0.0001 each, **Supplementary Figure S1**). This observation prompted us to determine whether associations between ANO7 expression and tumor phenotype were dependent on the ERG status. Separate analyses of ERG-negative and -positive cancers showed, however, that similarly strong associations with unfavorable tumor type (**Supplementary Tables S2** and **S3**) and patient outcome (*P* < 0.0001 each; **Figure 2B, 2C**) were seen in both subsets. Low ANO7 staining was also linked to high androgen receptor (AR) expression. This was seen in all cancers (*P* < 0.0001) and in ERG-negative cancers (*P* = 0.0002) but was not significant in ERG-positive cancers (**Figure 3**).

### Associations with chromosomal deletions and Ki67 labeling index

**Figure 4:**
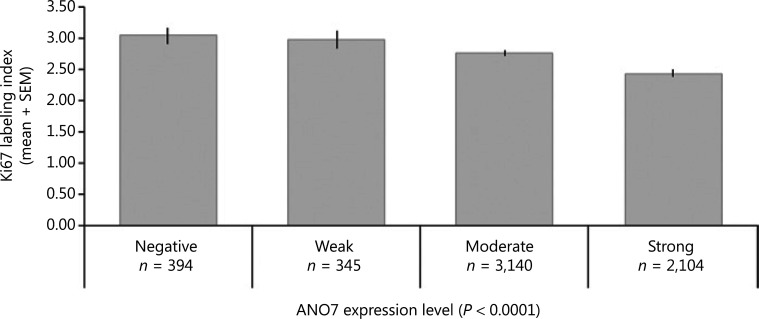
Anoctamin 7 (ANO7) staining and Ki67 labeling index.

For 9 of 11 analyzed chromosomal regions, ANO7 staining was weaker and more often negative in cases of deletion (**Supplementary Figure S2A**). In ERG-negative and ERG-positive cancers, a statistically significant difference was still seen for 5 of 11 and 8 of 11 analyzed deletions (*P* < 0.05 each, **Supplementary Figure S2B, S2C**). A particularly strong association was found between reduced ANO7 staining and the presence of *PTEN* (10q23) deletions, which was highly significant in all cancers as well as in ERG-negative and ERG-positive cancers (*P* < 0.0001 each). Reduced or lost ANO7 staining was significantly linked to increased cell proliferation as measured by Ki67LI (*P* < 0.0001; **Figure 4**). However, this association did not hold true in subsets of cancers with identical Gleason score.

### Multivariable analysis

The potential importance of ANO7 staining was analyzed in four different scenarios (**Table 2**, **Supplementary Table S4**). Scenario 1 evaluated all postoperatively available parameters including pathological tumor (pT), pathological lymph node (pN), surgical margin status, preoperative PSA value, and Gleason grade obtained on the prostatectomy specimen. In scenario 2, all postoperatively available parameters except pN were included. The rationale for this approach was that the indication and extent of lymph node dissection is not standardized in the surgical therapy of PCa and may introduce a bias toward high-grade cancers. Two additional scenarios were to model the preoperative situation as much as possible. Scenario 3 included ANO7 expression, preoperative PSA, clinical tumor stage (cT stage), and Gleason grade obtained on the prostatectomy specimen. As postoperative determination of a tumor’s Gleason grade is superior to the preoperatively determined Gleason grade (subjected to sampling errors and consequently under grading in more than one third of cases), this parameter was replaced by the preoperative Gleason grade obtained on the original biopsy in scenario 4. All these analyses identified ANO7 as a strong independent prognostic feature in the entire cohort and also in the subgroups of ERG-negative and -positive cancers (*P* < 0.0001 each). The striking prognostic role of ANO7 loss is also demonstrated by its prognostic relevance in multiple subgroups of cancers with identical traditional (3+4, 4+3, ≥ 8; *P* < 0.0005 each; **Supplementary Figure S3A**) and quantitative Gleason grade (3 of 7 groups, **Supplementary Figure S3B–S3H**). A striking prognostic impact of ANO7 loss was also seen in the subset of 982 *PTEN*-deleted cancers (*P* = 0.0033; **Figure 2E**).

**Table 2:** Hazard ratios (95% confidence intervals) for biochemical relapse after prostatectomy for established risk factors and ANO7 expression

Model	Analyzable (*n*)	Scenario 4	Scenario 3	Scenario 2	Scenario 1
Variable		9527	11,032	11,213	7214
Gleason grade biopsy	3+4 *vs.* ≤ 3+3	1.8 (1.6–2.0), *P* < 0.0001			
	4+3 *vs.* ≤ 3+3	3.0 (2.7–3.4), *P* < 0.0001			
	≥ 4+4 *vs.* ≤ 3+3	3.9 (3.4–4.4), *P* < 0.0001			
cT stage	T2a *vs.* T1c	1.3 (1.1–1.4), *P* < 0.0001	1.3 (1.1–1.4), *P* < 0.0001		
	T2b *vs.* T1c	1.9 (1.6–2.1), *P* < 0.0001	1.7 (1.5–1.9), *P* < 0.0001		
	T2c *vs.* T1c	1.9 (1.5–2.4), *P* < 0.0001	1.9 (1.5–2.4), *P* < 0.0001		
	T3a *vs.* T1c	1.3 (1.0–1.7), *P* = 0.0464	1.5 (1.2–2.0), *P* = 0.0011		
Preoperative PSA level	4–10 *vs.* < 4	1.6 (1.3–1.9), *P* < 0.0001	1.4 (1.2–1.7), *P* < 0.0001	1.3 (1.1–1.5), *P* = 0.0008	
	10–20 *vs.* < 4	2.4 (2.0–2.8), *P* < 0.0001	2.0 (1.7–2.4), *P* < 0.0001	1.7 (1.4–2.0), *P* < 0.0001	
	> 20 *vs.* < 4	3.8 (3.1–4.6), *P* < 0.0001	2.9 (2.4–3.4), *P* < 0.0001	2.0 (1.7–2.4), *P* < 0.0001	
ANO7 expression	Moderate *vs.* strong	1.3 (1.2–1.5), *P* < 0.0001	1.2 (1.1–1.3), *P* = 0.000	1.1 (1.0–1.2), *P* = 0.0373	1.1 (1.0–1.2), *P* = 0.0645
	Weak *vs.* strong	1.8 (1.6–2.1), *P* < 0.0001	1.5 (1.3–1.8), *P* < 0.0001	1.3 (1.1–1.5), *P* = 0.0003	1.3 (1.4–1.6), *P* = 0.0003
	Negative *vs.* strong	2.0 (1.7–2.3), *P* < 0.0001	1.6 (1.4–1.9), *P* < 0.0001	1.5 (1.3–1.7), *P* < 0.0001	1.4 (1.2–1.7), *P* < 0.0001
Gleason grade prostatectomy	3+4 *vs.* ≤ 3+3		2.9 (2.5–3.3), *P* < 0.0001	2.3 (2.0–2.7), *P* < 0.0001	2.1 (1.7–2.7), *P* < 0.0001
	4+3 *vs.* ≤ 3+3		7.3 (6.2–8.6), *P* < 0.0001	4.9 (4.1–5.8), *P* < 0.0001	4.3 (3.4–5.4), *P* < 0.0001
	3+4 Tert. 5 *vs.* ≤ 3+3		5.2 (4.1–6.6), *P* < 0.0001	3.5 (2.8–4.4), *P* < 0.0001	3.2 (2.4–4.2), *P* < 0.0001
	4+3 Tert. 5 *vs.* ≤ 3+3		11.3 (9.4–13.5), *P* < 0.0001	6.1 (5.1–7.4), *P* < 0.0001	5.1 (4.0–6.5), *P* < 0.0001
	≥ 4+4 *vs.* ≤ 3+3		14.0 (11.7–16.9), *P* < 0.0001	6.8 (5.6–8.3), *P* < 0.0001	5.5 (4.3–7.1), *P* < 0.0001
pT stage	T3a *vs.* T2			2.0 (1.8–2.2), *P* < 0.0001	2.0 (1.8–2.2), *P* < 0.0001
	T3b-4 *vs.* T2			3.3 (2.9–3.7), *P* < 0.0001	2.9 (2.5–3.3), *P* < 0.0001
Resection margin status	R1 *vs.* R0			1.4 (1.3–1.5), *P* < 0.0001	1.2 (1.1–1.4), *P* < 0.0001
Nodal stage	N+ *vs.* N0				1.5 (1.3–1.7), *P* < 0.0001

ANO7, anoctamin 7; cT, clinical tumor; pT, pathological tumor.

## Discussion

The present data identify reduced ANO7 expression as a strong and independent predictor of poor patient prognosis in PCa.

ANO7 was strongly expressed in normal prostate glands in our study. This fits well to earlier studies reporting 100% positivity in 10–44 samples of normal or benign prostate tissues^6,7,26^. Only a few studies had analyzed ANO7 expression in PCa before. Das et al.^6^ reported 91% positive PCa in a cohort of 126 patients. In two subsequent studies, Mohsenzadegan et al.^7,26^ analyzed 123–152 PCa and also reported reduced expression in a fraction of cancers as compared to normal epithelial cells. Overall, these data strongly suggest that ANO7 expression can be reduced or lost during PCa development or progression.

The comparison with clinical data in 13,594 successfully analyzed patients identifies reduced ANO7 as one of the strongest prognostic molecular features in PCa. This is not only demonstrated by the independent prognostic value of ANO7 staining in all applied models but also by its retained prognostic impact in cancers with comparable traditional or quantitative Gleason grade. In our patient cohort, where Gleason grading has been very thoroughly performed, most prognostic molecular parameters lose their prognostic role in cancers with homogeneous morphology^27–29^. A strong prognostic role of ANO7 loss in PCa is also in line with earlier data from Mohsenzadegan et al.^7,26^ who reported an inverse correlation between ANO7 staining intensity and tumor stage or Gleason score. We cannot explain why a recent meta-analysis of ANO7 mRNA expression data on 289 tumors obtained from The Cancer Genome Atlas project database^8^ found a correlation between increased ANO7 expression and early biochemical relapse^9^. It cannot be excluded that mRNA expression does not always translate in immunohistochemically detectable ANO7 protein, and that true differences exist between the prognostic value of ANO7 mRNA and protein analysis. Alternatively, technical issues such as a variable contamination with ANO7-positive normal glandular cells or ANO7-negative stromal cells, a possible selection bias for larger advanced cancers that can be more easily used for next-generation sequencing, or the comparatively low number of patients may account for these discrepant findings.

The reduction of ANO7 expression from well-differentiated to poorly differentiated prostate cells may suggest its total expression loss in aggressive PCa. This indicates that ANO7 could be a surrogate of molecular driver of tumor aggression in PCa that is yet to be discovered. The physiologic function of ANO7 is not sufficiently known. A Medline search on March 28, 2019 identified fewer than 20 publications on ANO7 or NGEP. Being an ion channel protein, ANO7 may influence levels and compartmentalization of intracellular calcium^30^. Intracellular calcium levels can affect the activity of genes that are relevant for cancer development and progression^30^. Ion channel proteins with a well-accepted role in cancer biology, for example, include potassium channels, Cl^−^ channels, and Na^+^ channels^31,32^. Some other anoctamins have been earlier linked to cancer biology. For example, anoctamin 1 is particularly expressed in gastrointestinal stromal tumors and head and neck squamous cell carcinomas, where it contributes to cell proliferation, poor prognosis, and metastasis^33,34^. A splice variant of anoctamin 6 has been associated with poor prognosis in breast cancer^35^.

It is also possible that the striking prognostic impact of ANO7 loss is not related to its – so far unknown – function. We had recently found a strong link between reduced cellular expression of PSA and poor PCa prognosis^36^. PSA and ANO7 share a complete specificity for prostate epithelial cells and the very high level of expression in these cells. Although some authors have speculated on a possible cancer preventive role of PSA, this has never been proven^37^. The main physiological role of PSA is semen liquidification. The prognostic impact of reduced PSA was very strong, comparable to the role of ANO7 loss, and also found in *PTEN*-deleted cancers. For PSA, we had speculated that a measurable loss of one of the most important cellular proteins might represent a subtle sign of cellular dedifferentiation. In analogy to the PSA situation, we consider it possible that ANO7 is another important component of prostate epithelial cells without a cancer-specific role and that a reduced expression might indicate cellular dedifferentiation.

The abundant data available from earlier studies using our PCa cohort enabled us to compare ANO7 expression with several important molecular features. *TMPRSS2:ERG* fusions occur in about 50% of PCa resulting in permanent overexpression of the transcription factor ERG. This overexpression modulates the expression of more than 1600 genes but lacks prognostic relevance by itself. Our data suggest that loss of ANO7 expression is ERG dependent as ANO7 protein levels were clearly lower in ERG-positive than in ERG-negative cancers. Such inverse associations found by IHC are particularly certain to represent true associations. This observation thus provides further evidence for the validity of our experimental procedures. A mild positive association between immunohistochemically determined parameters could always be due to a fraction of samples that are non-reactive to IHC resulting in “negative” staining results for all parameters measured. The same might hold true also for the inverse association between ANO7 expression and AR positivity. That this weak association was statistically significant only in the subset of ERG-negative cancers might be first of all related to the strong link between AR and ERG, which might obscure less strong interactions. However, AR does not belong to the more than 100 transcription factors that are known to impact ANO7 expression^37^.

Chromosomal deletions represent the most common recurrent genomic alterations in PCa after *TMPRSS2:ERG* fusions. That most deletions were significantly associated with reduced ANO7 expression could suggest a role of ANO7 in mechanisms regulating genomic instability. Alternatively, these findings would also be consistent with the notion of reduced ANO7 expression representing a dedifferentiation surrogate. The particularly low levels of ANO7 expression in *PTEN*-deleted cancers are consistent with a direct or indirect functional interaction between ANO7 and the PTEN/AKT pathway. That the interaction with PTEN is not responsible for the prognostic impact of reduced ANO7 expression is demonstrated by its retained prognostic role in *PTEN*-deleted cancers. This is unusual and further argues for a particularly strong prognostic role of ANO7 expression loss in PCa. *PTEN* deletions are strongly linked to poor prognosis in PCa. Many prognostic features fail to further stratify patient outcome in molecular subgroups that are already defined by *PTEN* deletion^38^. Several studies had suggested an impact of ANO1 on tumor cell proliferation^39–41^. In our patients, tumor cell proliferation was only marginally linked to ANO7 expression and significant associations could often not be found in homogeneous subgroups. We consider it thus unlikely that ANO7 exerts a potential tumor suppressive function through impacting tumor cell proliferation. Thus, we favor the notion that ANO7 loss indicates loss of polar orientation in prostate epithelial cells as a form of dedifferentiation.

The strong independent prognostic role found for ANO7 expression in this study suggests that measuring this protein could result in useful prognostic information for PCa patients. Further studies using different antibodies and independent patient cohorts potentially also including patients treated with radiotherapy or radiotherapy and hormonal therapy, as well as premalignant prostate conditions, would be helpful to confirm the validity of ANO7 as a routine diagnosis marker in prostate and potentially even other human cancer types. If immunohistochemical analyses are meant to provide clinically relevant information to trigger treatment decisions, it will be critical to apply highly reproducible assays. The advent of multiplex IHC enabling the simultaneous application of multiple antibodies and a computerized quantification of individual proteins may assist in making this possible.

In summary, reduced ANO7 expression is tightly linked to an unfavorable disease course in PCa. The statistical independence from pre- and postoperatively available parameters argues for a possible diagnostic application of ANO7 measurement, either alone or combined with other parameters.
