# Supplementary material for: Reduced anoctamin 7 (ANO7) expression is a strong and independent predictor of poor prognosis in prostate cancer
Source: Cancer Biol Med. 2021 Feb 15;18(1):245–55. doi: 10.20892/j.issn.2095-3941.2019.0324 (PMC7877177; doi:10.20892/j.issn.2095-3941.2019.0324)
Supplement: Supplementary file 1 [file cbm-18-245-s001.pdf]

# Supplementary materials

**Table S1** Composition of the prostate prognosis tissue microarray

| | No. of patients (%) | |
|---|---|---|
| | Study cohort on TMA* | Biochemical relapse among categories |
| Follow-up | | |
| *n* | 14,464 | 3,612 (25%) |
| Mean/median (months) | 56/48 | – |
| Age (years) | | |
| ≤ 50 | 433 | 66 (15.2%) |
| 51–59 | 4,341 | 839 (19.3%) |
| 60–69 | 9,977 | 2,073 (20.8%) |
| ≥ 70 | 2,936 | 634 (21.6%) |
| Pretreatment PSA (ng/mL) | | |
| < 4 | 2,225 | 313 (14.1%) |
| 4–10 | 10,520 | 1,696 (16.1%) |
| 10–20 | 3,662 | 1,043 (28.5%) |
| > 20 | 123 | 545 (44.3%) |
| pT stage (AJCC 2002) | | |
| pT2 | 11,518 | 1,212 (10.5%) |
| pT3a | 3,842 | 1,121 (29.2%) |
| pT3b | 2,233 | 1,213 (54.3%) |
| pT4 | 85 | 63 (74.1%) |
| Gleason grade | | |
| ≤ 3+3 | 3,570 | 264 (7.4%) |
| 3+4 | 9,336 | 1,436 (15.4%) |
| 3+4 Tert.5 | 1,697 | 165 (9.7%) |
| 4+3 | 2,903 | 683 (23.5%) |
| 4+3 Tert.5 | 1,187 | 487 (41%) |
| ≥ 4+4 | 999 | 531 (53.2%) |
| pN stage | | |
| pN0 | 10,636 | 2,243 (21.1%) |
| pN+ | 1,255 | 700 (55.8%) |
| Surgical margin | | |
| Negative | 14,297 | 2,307 (16.1%) |
| Positive | 3,388 | 1,304 (38.5%) |

AJCC, American Joint Committee on Cancer; cT, clinical tumor; pT, pathological tumor; pN, pathological lymph node; PSA, prostate-specific antigen; TMA, tissue microarray. *Numbers do not always add up to 17,747 in the different categories because of cases with missing data.

**Table S2** ANO7 expression and prostate cancer phenotype in the ERG-negative subset

| Parameter | *n* | Fraction of ANO7 expression (%) | | | | *P* |
|---|---|---|---|---|---|---|
| | | Negative | Weak | Moderate | Strong | |
| All cancers | 4915 | 7.0 | 5.3 | 44.5 | 43.2 | |
| Tumor stage | | | | | | < 0.0001 |
| pT2 | 8889 | 5.5 | 7.0 | 47.4 | 40.2 | |
| pT3a | 2984 | 9.8 | 11.0 | 52.1 | 27.1 | |
| pT3b-pT4 | 1668 | 14.5 | 18.5 | 49.6 | 17.3 | |
| Gleason grade | | | | | | < 0.0001 |
| ≤ 3+3 | 1002 | 3.7 | 3.6 | 40.4 | 52.3 | |
| 3+4 | 2670 | 5.1 | 4.5 | 44.0 | 46.3 | |
| 3+4 Tert.5 | 216 | 5.6 | 7.4 | 41.7 | 45.4 | |
| 4+3 | 509 | 15.1 | 6.3 | 48.7 | 29.9 | |
| 4+3 Tert.5 | 282 | 11.3 | 8.2 | 54.3 | 26.2 | |
| ≥ 4+4 | 233 | 20.6 | 13.7 | 50.2 | 15.5 | |
| Quantitative Gleason grade | | | | | | < 0.0001 |
| ≤ 3+3 | 1002 | 3.7 | 3.6 | 40.4 | 52.3 | |
| 3+4 ≤ 5% | 730 | 4.5 | 3.8 | 41.1 | 50.5 | |
| 3+4 (6%–10%) | 697 | 3.4 | 3.6 | 43.3 | 49.6 | |
| 3+4 (11%–20%) | 595 | 4.5 | 4.7 | 45.5 | 45.2 | |
| 3+4 (21%–30%) | 291 | 7.2 | 7.6 | 46.7 | 38.5 | |
| 3+4 (31%–49%) | 261 | 8.8 | 5.4 | 46.7 | 39.1 | |
| 3+4 Tert.5 | 216 | 5.6 | 7.4 | 41.7 | 45.4 | |
| 4+3 (50%–60%) | 226 | 11.9 | 4.9 | 44.7 | 38.5 | |
| 4+3 Tert.5 | 282 | 11.3 | 8.2 | 54.3 | 26.2 | |
| 4+3 (61%–100%) | 235 | 16.6 | 7.2 | 53.2 | 23.0 | |
| ≥ 4+4 | 209 | 20.6 | 12.0 | 50.7 | 16.7 | |
| Lymph node metastasis | | | | | | < 0.0001 |
| N0 | 2876 | 7.0 | 5.5 | 45.8 | 41.7 | |
| N+ | 248 | 19.8 | 13.3 | 46.4 | 20.6 | |
| Preoperative PSA level (ng/mL) | | | | | | < 0.0001 |
| < 4 | 523 | 8.0 | 6.1 | 44.9 | 40.9 | |
| 4–10 | 2936 | 5.8 | 4.5 | 43.4 | 46.4 | |
| 10–20 | 1082 | 8.0 | 5.3 | 47.4 | 39.3 | |
| > 20 | 349 | 12.0 | 10.9 | 45.6 | 31.5 | |
| Surgical margin | | | | | | < 0.0001 |
| Negative | 3951 | 6.1 | 4.9 | 44.5 | 44.5 | |
| Positive | 952 | 10.6 | 6.7 | 44.9 | 37.8 | |

ANO7, anoctamin 7; ERG, erythroblast transformation-specific-related gene; PSA, prostate-specific antigen.

**Table S3** ANO7 expression and prostate cancer phenotype in the ERG-positive subset

| Parameter | *n* | Fraction of ANO7 expression (%) | | | | *P* |
|---|---|---|---|---|---|---|
| | | Negative | Weak | Moderate | Strong | |
| All cancers | 3895 | 7.4 | 7.4 | 60.3 | 24.9 | |
| Tumor stage | | | | | | < 0.0001 |
| pT2 | 2367 | 5.1 | 5.1 | 58.9 | 31.0 | |
| pT3a | 1027 | 10.3 | 9.2 | 63.9 | 16.7 | |
| pT3b-pT4 | 485 | 12.8 | 14.2 | 59.8 | 13.2 | |
| Gleason grade | | | | | | < 0.0001 |
| ≤ 3+3 | 815 | 4.3 | 2.8 | 58.4 | 34.5 | |
| 3+4 | 2261 | 6.0 | 6.6 | 62.2 | 25.2 | |
| 3+4 Tert.5 | 116 | 10.3 | 10.3 | 59.5 | 19.8 | |
| 4+3 | 377 | 14.6 | 12.7 | 57.3 | 15.4 | |
| 4+3 Tert.5 | 205 | 10.7 | 16.1 | 60.0 | 13.2 | |
| ≥ 4+4 | 118 | 24.6 | 16.9 | 47.5 | 11.0 | |
| Quantitative Gleason grade | | | | | | < 0.0001 |
| ≤ 3+3 | 815 | 4.3 | 2.8 | 58.4 | 34.5 | |
| 3+4 ≤ 5% | 569 | 2.8 | 4.2 | 57.8 | 35.1 | |
| 3+4 (6%–10%) | 600 | 5.3 | 7.2 | 62.8 | 24.7 | |
| 3+4 (11%–20%) | 492 | 5.7 | 7.1 | 66.1 | 21.1 | |
| 3+4 (21%–30%) | 283 | 7.4 | 8.1 | 63.3 | 21.2 | |
| 3+4 (31%–49%) | 197 | 11.2 | 6.1 | 67.5 | 15.2 | |
| 3+4 Tert.5 | 116 | 10.3 | 10.3 | 59.5 | 19.8 | |
| 4+3 (50%–60%) | 173 | 6.4 | 12.7 | 59.0 | 22.0 | |
| 4+3 Tert.5 | 205 | 10.7 | 16.1 | 60.0 | 13.2 | |
| 4+3 (61%–100%) | 163 | 20.2 | 14.1 | 54.6 | 11.0 | |
| ≥ 4+4 | 98 | 21.4 | 16.3 | 52.0 | 10.2 | |
| Lymph node metastasis | | | | | | < 0.0001 |
| N0 | 2248 | 9.2 | 7.9 | 58.9 | 24.1 | |
| N+ | 220 | 12.7 | 15.9 | 59.5 | 11.8 | |
| Preoperative PSA level (ng/mL) | | | | | | < 0.0001 |
| < 4 | 530 | 8.3 | 5.5 | 62.3 | 24.0 | |
| 4–10 | 2409 | 6.0 | 6.9 | 59.5 | 27.6 | |
| 10–20 | 694 | 9.8 | 8.1 | 62.0 | 20.2 | |
| > 20 | 233 | 12.9 | 12.9 | 58.4 | 15.9 | |
| Surgical margin | | | | | | 0.0001 |
| Negative | 3072 | 7.2 | 6.8 | 59.5 | 26.5 | |
| Positive | 806 | 8.2 | 9.2 | 63.3 | 19.4 | |

ANO7, anoctamin 7; ERG, erythroblast transformation-specific-related gene; PSA, prostate-specific antigen.

**Table S4** Multivariate Cox regression analysis with established prognostic parameters and the ANO7 expression in all prostate cancers, the ERG-negative, and ERG-positive subsets

| Tumor subset | Scenario | *n* analyzable | *P*-value | | | | | | | |
|---|---|---|---|---|---|---|---|---|---|---|
| | | | Preoperative PSA level | pT stage | cT stage | Gleason grade prostatectomy | Gleason grade biopsy | pN stage | R stage | ANO7 expression |
| All cancers | 1 | 7173 | < 0.0001 | < 0.0001 | – | < 0.0001 | – | < 0.0001 | < 0.0001 | < 0.0001 |
| | 2 | 11,141 | < 0.0001 | < 0.0001 | – | < 0.0001 | – | - | < 0.0001 | < 0.0001 |
| | 3 | 10,965 | < 0.0001 | – | < 0.0001 | < 0.0001 | – | – | – | < 0.0001 |
| | 4 | 9527 | < 0.0001 | – | < 0.0001 | – | < 0.0001 | – | – | < 0.0001 |
| ERG-negative cancers | 1 | 2877 | 0.0054 | < 0.0001 | – | < 0.0001 | – | 0.0004 | 0.00537 | 0.0032 |
| | 2 | 4518 | < 0.0001 | < 0.0001 | – | < 0.0001 | – | – | 0.0004 | 0.0028 |
| | 3 | 4474 | < 0.0001 | – | < 0.0001 | < 0.0001 | – | – | – | 0.0002 |
| | 4 | 4404 | < 0.0001 | – | < 0.0001 | – | < 0.0001 | – | – | < 0.0001 |
| ERG-positive cancers | 1 | 2274 | 0.0016 | < 0.0001 | – | < 0.0001 | – | 0.0166 | < 0.0001 | 0.0002 |
| | 2 | 3567 | < 0.0001 | < 0.0001 | – | < 0.0001 | – | – | < 0.0001 | 0.0001 |
| | 3 | 3511 | < 0.0001 | – | < 0.0001 | < 0.0001 | – | – | – | < 0.0001 |
| | 4 | 3455 | < 0.0001 | – | < 0.0001 | – | < 0.0001 | – | – | < 0.0001 |

ANO7, anoctamin 7; ERG, erythroblast transformation-specific-related gene. Scenario 1 includes all postoperatively available parameters: pathological tumor (pT) stage, lymph node (pN), surgical margin (R) status, preoperative prostate-specific antigen (PSA) value, and Gleason grade obtained after the morphological evaluation of the entire resected prostate. Scenario 2 excluded the nodal status from analysis. Scenario 3 included preoperative PSA, clinical tumor (cT) stage, and Gleason grade obtained on the prostatectomy specimen. In scenario 4, the preoperative Gleason grade obtained on the original biopsy was combined with preoperative PSA and cT stage.

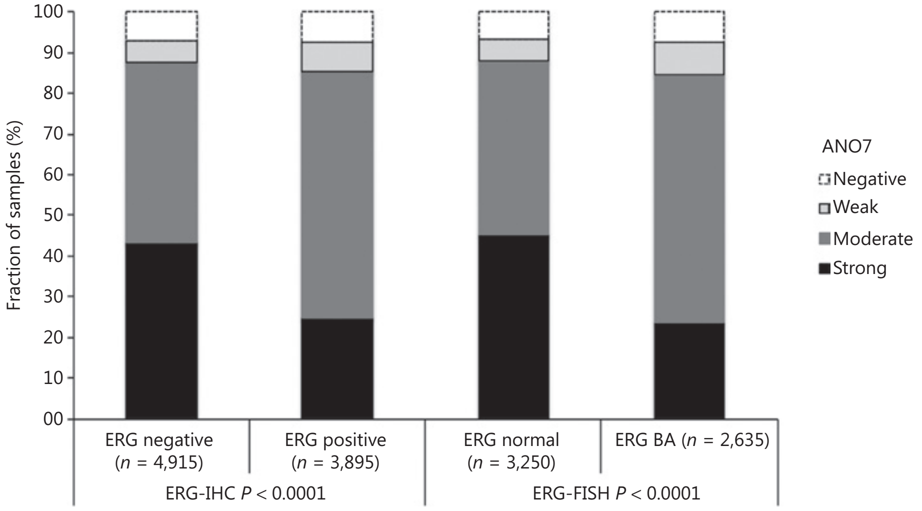


**Figure S1** Anoctamin 7 (ANO7) expression and erythroblast transformation-specific-related gene (ERG) status [immunohistochemistry (IHC)/fluorescence *in situ* hybridization (FISH)].

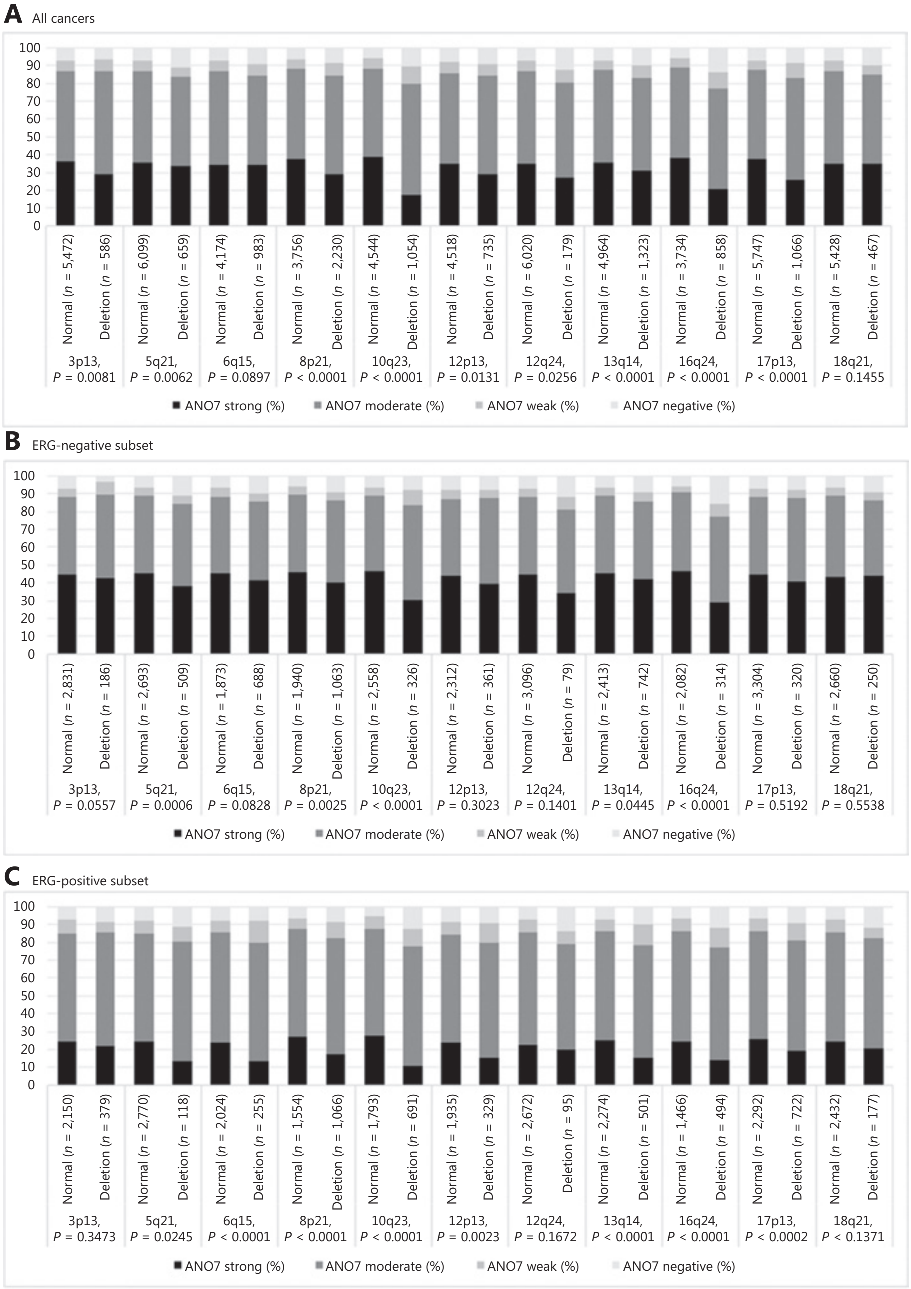


**Figure S2** Anoctamin 7 (ANO7) staining and genomic deletions.

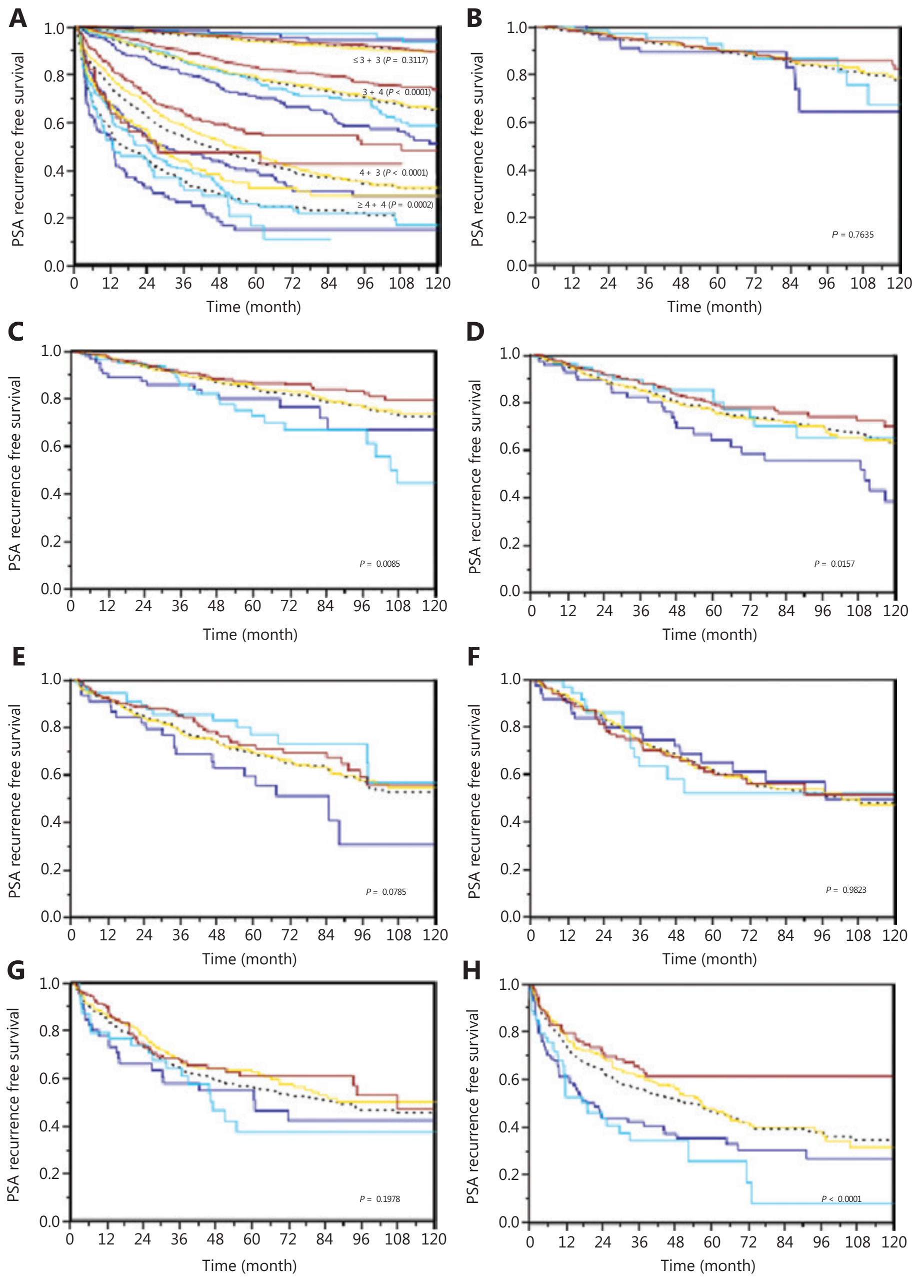


**Figure S3** Prognostic impact of anoctamin 7 (ANO7) expression defined by the Gleason score. (A) Impact of ANO7 expression as compared to the classical Gleason score categories; (B–H) as compared to the quantitative Gleason score categories defined by (B) ≤ 5%, (C) 6%–10%, (D) 11%–20%, (E) 21%–30%, (F) 31%–49 %, (G) 50%–60%, and (H) ≥ 61% Gleason 4 patterns. Dotted line shows the reference for the respective Gleason category.
